# Increase in physical activity is associated with an increase in sleep efficiency, but not with improvement in symptoms of PTSD: analysis of longitudinal data in trauma-affected refugees

**DOI:** 10.1186/s44167-024-00046-8

**Published:** 2024-02-15

**Authors:** Hinuga Sandahl, Mette Korshøj, Ole Steen Mortensen, Jessica Carlsson

**Affiliations:** 1 Competence Centre for Transcultural Psychiatry, Mental Health Center Ballerup, Copenhagen University Hospital– Mental Health Services CPH , Ballerup, Denmark; 2 Department of Occupational and Social Medicine, Holbaek Hospital, part of University of Copenhagen, Holbaek, Denmark; 3 Department of Public Health, Section of Social Medicine, University of Copenhagen, Copenhagen, Denmark; 4 Dept of Clinical Medicine, University of Copenhagen, Copenhagen, Denmark

**Keywords:** Physical activity, Sleep quality, PTSD, Quality of life, Refugees

## Abstract

**Background:**

In trauma-affected refugees with posttraumatic stress disorder (PTSD), research on physical activity is scarce. Knowing more about the relation between physical activity and PTSD symptoms may provide insight into physical activity as a possible target in the treatment of PTSD. The aim of the present study was to examine whether baseline and change in level of physical activity from baseline to end of treatment were related to, respectively, baseline and change in PTSD symptoms, quality of life, sleep quality, and sleep efficiency in trauma-affected refugees.

**Methods:**

Longitudinal data from a randomized controlled trial were analysed with multiple linear regression. Level of physical activity and sleep efficiency were measured with actigraphy and symptoms of PTSD, sleep quality, and quality of life were measured with self-report questionnaires.

**Results:**

A higher level of physical activity was significantly associated with better baseline sleep quality, borderline associated with quality of life, but not with symptoms of PTSD, or sleep efficiency. Furthermore, an increase in level of physical activity was significantly associated with improvement in sleep efficiency. Change in level of physical activity was not significantly associated with improvement in PTSD symptoms, quality of life, or sleep quality.

**Conclusion:**

The novelty of the current study lies in the finding of no relation between a change in level of physical activity and a change in symptoms of PTSD. The results point to a complex relation between sleep, physical activity and PTSD and point towards a need for studies on these relations to provide effective interventions in trauma-affected refugees.

**Trial registration:**

ClinicalTrials.gov ID (NCT02761161), April 27, 2016.

## Background

Globally, the number of refugees has increased during the last decade reaching an unprecedented 35.3 million by the end of 2022 [1]. Furthermore, the Russian invasion of Ukraine has forced over 8 million refugees to flee from Ukraine [2]. A refugee is defined as an individual who “owing to well-founded fear of being persecuted for reasons of race, religion, nationality, membership of a particular social group or political opinion, is outside the country of his nationality and is unable or, owing to such fear, is unwilling to avail himself of the protection of that country” [3]. Refugees are at high risk of posttraumatic stress disorder (PTSD) as a consequence of traumas, such as imprisonment, torture, combat experience, violent sexual assault, and the violent death of relatives. Post-migration risk factors, such as separation from family members and poor socioeconomic conditions, unemployment, and social isolation may contribute to the development of mental disorders. PTSD prevalence in refugees is estimated to be around 30% [4, 5].

With the rapid increase in the number of refugees during the last decade and the absolute number of refugees with PTSD in high-income countries increasing, appropriate and effective treatment interventions in mental health care services are highly warranted [6–10]. Several meta-analyses have been published on psychosocial interventions for trauma-affected refugees suggesting that interventions such as Cognitive Behavioral Therapy (CBT), Narrative Exposure Therapy, and Eye Movement Desensitization and Reprocessing can reduce symptoms of PTSD. However, analyses also conclude that considerable heterogeneity exists between studies indicating that the efficacy may vary significantly and that significant numbers of refugees continue to show symptoms at end of treatment [6, 7, 11–16].

Poor sleep quality —including difficulties initiating or maintaining sleep and nightmares are part of the diagnostic criteria for PTSD [10, 17]. However, standard PTSD treatment has not traditionally addressed sleep quality, and poor sleep quality often persists after PTSD treatment [17]. In attempts to enhance treatment outcomes, different treatment modalities have been examined focusing on different aspects of PTSD symptomatology. Meta-analyses and reviews find that physical activity and exercise training show promising results in the treatment of PTSD symptoms including sleep quality in non-refugee populations with PTSD [18–21].

In the specific population of refugees, research on physical activity is scarce. Previously, a randomized controlled trial (RCT) has examined the effects of physical activity in trauma-affected refugees and another RCT has examined the effect of group-based physiotherapy intervention in trauma-affected refugees, both identifying no significant effect on symptoms of PTSD [22, 23]. However, a cross-sectional study with a large sample of asylum seekers has identified low level of self-reported physical activity to be associated with higher PTSD symptom severity [24].

Several studies with non-refugee populations have examined the relationship between PTSD symptom severity and level of physical activity, in cross-sectional studies, delineating mixed results [25]. A review found half of the included studies to report a significant association between PTSD and physical activity, and the other half reported no significant associations between PTSD and physical activity [25]. The studies reporting a significant association identified that participants with PTSD reported significantly less vigorous physical activity, more infrequent physical activity, and that fewer participated in weekly physical activity [26–28]. Furthermore, increased engagement in vigorous physical activity was significantly associated with reduced odds of having new-onset symptoms and/or persistent PTSD symptoms [26].

Most studies on physical activity and symptoms of PTSD rely on self-reported data and are cross-sectional in design. Studies using technical measures for physical activity and longitudinal studies are called for [25, 29]. There is a lack of studies on the relationship between changes in the level of physical activity and PTSD symptoms in trauma-affected populations, in general terms, and no such studies in the specific population of refugees. Furthermore, previous research has suggested a role of physical activity in relation to both self-reported aspects such as quality of life, and sleep quality and technical measures concerning sleep, such as sleep efficiency [20, 29–31]. However, to the best of our knowledge, these relations have not been examined in a population of trauma-affected refugees. Knowing more about the relation between change in the level of physical activity over time and treatment response for PTSD symptoms, quality of life, quality of sleep, and sleep efficiency, may provide insight on the relation between physical activity, sleep and PTSD, in general, and in the specific population of trauma-affected refugees.

The aim of the present study was therefore to examine whether:

- Baseline level of physical activity was related to baseline PTSD symptoms, quality of life, sleep quality, and sleep efficiency in a population of trauma-affected refugees.
- change in the level of physical activity from baseline to end of treatment was related to changes in PTSD symptoms, quality of life, sleep quality, and sleep efficiency from baseline to end of treatment in a population of trauma-affected refugees.

## Methods

### Study design and participants

We analysed data from refugees with PTSD having participated in a RCT performed at the Competence Centre for Transcultural Psychiatry (CTP) in the period 2016–2019 [32, 33]. The study design and the data collection methods have been described in detail elsewhere [32, 33].

Participants were adult refugees diagnosed with PTSD who provided written informed consent for study participation. Inclusion criteria were PTSD diagnosis according to ICD-10 determined in a clinical interview, poor sleep quality measured as a score > 8 on the Pittsburgh Sleep Quality Index, and nightmares measured as a score ≥ “a little” on the Harvard Trauma Questionnaire nightmare item. Exclusion criteria were severe psychotic disorder, a current alcohol or drug use disorder, a neurodegenerative disorder or an acute need of admission to a psychiatric inpatient facility.

The overall aim of the RCT was to estimate treatment effects in trauma-affected refugees. The participants were randomized to one of four intervention groups. All four groups received treatment-as-usual (TAU) consisting of a 10–12 months bio-psycho-social interdisciplinary treatment approach, including manual-based CBT, physiotherapy, pharmacological treatment according to standard and best clinical practice in the field (sertraline is first-choice antidepressant, venlafaxine second-choice antidepressant), psychoeducation, and social counselling within a frame of weekly sessions with a physician, a physiotherapist, a social counsellor, or psychologist.

One group received solely TAU to serve as a control group, while the three remaining groups were active treatment groups, receiving add-on treatment with either Imagery Rehearsal Therapy (IRT) (CBT focusing on nightmares) and/or mianserin (a sedative antidepressant), or a combination of both. The RCT identified no between-group differences in symptoms of PTSD, sleep quality or quality of life at baseline, end of treatment or from baseline to end of treatment [32, 33], and the current study examined the complete sample across intervention groups regardless of which treatment the participants received.

### Measures

In an initial assessment interview sociodemographic information was collected, including among other factors age, level of education and affiliation to the labour market. Baseline status and treatment response was evaluated using both self-administered rating scales and observer ratings. Symptoms of PTSD were assessed with the self-reported Harvard Trauma Questionnaire (HTQ), consisting of 16 items with a score range from 1 to 4 [34, 35]. HTQ is the most prevalent scale for evaluating PTSD symptoms in refugees. The HTQ has been developed in refugee research and the scale is perceived as reliable in a clinical refugee sample [36, 37]. Quality of life was measured by self-report on the WHO-5, consisting of five questions with a score range from 0 to 5 [38]. Sleep quality was measured on the Pittsburgh Sleep Quality Index (PSQI), consisting of 19 items and measuring seven components of Sleep. The component scores each have a range of 0–3 points and they are added to yield one global PSQI score with a score range from 0 to 21 [39, 40].

### Actigraphic assessment

The participants wore an Actiwatch Spectrum (Philips Respironics) device on their non-dominant wrist for 24 h a day for 14 consecutive days at baseline and at end of treatment. An Actiwatch measures wrist movement, and can be used to measure physical activity and sleep patterns [41, 42].

Activity counts were logged for 30-second epochs and stored in Actiware (version 6.0.9, Respironics, Murrysville, PA, USA) format files. For the analyses, data were processed to calculate activity per minute, hour, and 24 h. Actigraphy data were used to calculate the mean activity score per hour, during the most active 10-hour period (M10, counts/hour) per 24-h day to represent the mean level of daytime physical activity [41–43]. Furthermore, Actiware Version 6.0.9 Philips Respioronics 2020 was used to analyze sleep data. An automatic algorithm determined sleep and wake states and thus calculated sleep efficiency (SE).

Actigraphy data were excluded for participants with fewer than 7 days of recording to increase the reliability of the actigraphy measures [44]. Actigraphy data for participants with a minimum of 7 days’ measurements, each day with a minimum of 10 h of recording during wake time and a maximum of 1-hour non-recording during sleep time, for any of the recorded 7 days were included. All actograms were visually screened for sufficient wear time before being submitted for analysis. The data were cleaned manually by the first author according to prespecified editing rules. Event markers, activity counts, diary information and light input were used to adjust the start and end time points of the rest intervals, if necessary. Baseline actigraphy measures were included for 118 participants and baseline to end of treatment actigraphy measures were included for 54 participants.

Differences in the level of symptoms and sociodemographic characteristics were tested for between participants with and without actigraphy measures and between participants with and without end of treatment scores. We identified no significant differences in the level of symptoms or sociodemographic characteristics and missing actigraphy data were deemed at random.

### Statistical analysis

To describe the sample characteristics, means, and standard deviations (SD) were calculated for continuous variables, and percentages were calculated for categorical variables. Mean rating scores were calculated for baseline and end of treatment measures, and significant baseline to end of treatment differences were tested for with paired t-test.

A baseline to end of treatment variable was created displaying the difference in score between baseline score and end of treatment score.

In a multiple linear regression model, we tested whether the baseline level of daytime physical activity (exposure variable) was associated with baseline severity of PTSD, quality of life, sleep quality, and sleep efficiency (dependent variables). Baseline scores corresponding to the dependent variable, age, and sex were included as confounder variables.

In a multiple linear regression model, we tested whether change in the level of physical activity (exposure variable) was associated with change in the severity of PTSD, quality of life, sleep quality, and sleep efficiency. Baseline scores on the corresponding dependent variable, age, and sex were included as confounder variables.

We performed multiple linear regression analyses using STATA’s SEM procedure, applying a Full Information Maximum Likelihood (FIML) analysis to handle missing data. FIML estimates a likelihood function for each individual based on the present variables in order to use all available data, including data for participants with missing end of treatment scores.

To check assumptions of linearity, a scatterplot of the included variables was plotted with a superimposed regression line. Visual inspection of these plots indicated a linear relationship between the continuous variables and no outliers of importance. Independence of errors, homoscedasticity, and normality of residuals was met to a reasonable degree.

Statistical analyses were conducted using Stata version 14.1 for windows.

## Results

The study included 219 participants with a mean age of 44.4 years (SD 10.4). There were 110 (50%) female participants. Sociodemographic characteristics are presented in Table 1.

**Table 1:** Sociodemographic characteristics of sample (*N* = 219, except where noted)

	Mean (SD)
Age (years)	44.44 (10.40)
Years since arrival in Denmark (*n* = 211) *	13.33 (9.55)
	***n*** **(%)**
Female	110 (50)
Male	109 (50)
Country of origin (*n* = 207)*	
Afghanistan	26 (13)
Iran	19 (9)
Iraq	54 (26)
Lebanon	15 (7)
Syria	58 (28)
Other	35 (17)
Psychosocial status	
Needing translator during medical doctor sessions (*n* = 189)*	119 (63)
Affiliation to the labour market / studying (*n* = 183)*	66 (36)
Income from labour (*n* = 196)*	13 (7)
Living alone all the time (*n* = 198)*	28 (14)
Having children of < 18 years of age (*n* = 160)*	130 (80)
Education > 10 years from home country (*n* = 165)*	86 (52)
Work experience in Denmark (*n* = 197)*	96 (48)
Smoking (*n* = 185)*	67 (36)
Diagnoses (ICD-10)	
PTSD	219 (100)
Depression	157 (72)

*SD*, standard deviation

ICD-10, International Classification of Disease

PTSD, posttraumatic stress disorder

*Data not available for all randomized participants

Mean scores at baseline, end of treatment, and differences from baseline to end of treatment for PTSD symptoms, quality of life, sleep quality, physical activity, and sleep efficiency are presented in Table 2. We found a significant improvement in PTSD symptoms, quality of life, sleep quality, and a significant decrease in sleep efficiency. No differences were seen in the level of physical activity. However, the standard deviation for change score for level of physical activity is quite large with values spread out from the mean, exhibiting large variation in change in level of physical activity.

**Table 2:** Mean scores at baseline and end of treatment for self-reported PTSD symptoms (HTQ), quality of sleep (PSQI), quality of life (WHO-5), actigraphy-derived activity count (M10) and sleep efficiency (SE)

Rating	Mean score (SD) baseline	Mean score (SD) end of treatment	Difference baseline-end of treatment	*p*-value difference baseline-end of treatment
Self-reported				
HTQ, *n* = 159	3.14 (0.41)	2.95 (0.62)	-0.18 (0.59)	< 0.0001*
PSQI, *n* = 154	16.35 (2.69)	14.53 (4.29)	-1.82 (4.27)	< 0.0001*
WHO-5, *n* = 146	17.25 (16.39)	25.18 (23.07)	7.94 (21.68)	< 0.0001*
Actigraphy measures				
M10, *n* = 54	267.13 (85.65)	268.43 (89.83)	1.30 (63.18)	0.880
SE, *n* = 55	80.80 (6.88)	79.38 (6.94)	-1.41 (5.09)	0.045*

Raw baseline and end of treatment scores and score differences for ratings for participants for which both baseline and end of treatment scores are available

SD = Standard deviation

* = Statistically significant improvement

HTQ, Harvard Trauma Questionnaire: 1–4 (1 best score), PSQI Pittsburgh Sleep Quality Index: 1–21 (1 best score), WHO-5 Well Being Index: 1-100 (100 best score), M10, mean activity score of the most active 10-hour period, measured in counts/hour; SE Sleep efficiency, measured in %

Table 3 presents the results of the multiple linear regression analyses on baseline values adjusted for age and sex.

**Table 3:** Multiple regression analyses of baseline level of physical activity on PTSD symptoms (HTQ), quality of sleep (PSQI), quality of life (WHO-5), and sleep efficiency (SE) adjusted for age and sex

	HTQ					PSQI					SE					WHO-5				
	β	95% CI for β		*p*	R^2^	β	95% CI for β		*p*	R^2^	β	95% CI for β		*p*	R^2^	β	95% CI for β		*p*	R^2^
		*LL*	*UL*				*LL*	*UL*				*LL*	*UL*				*LL*	*UL*		
					0.04					0.14					0.04					0.06
**Age**	0.08	-0.06	0.23	0.272		0.05	-0.10	0.19	0.534		0.01	-0.18	0.19	0.359		-0.12	-0.27	0.04	0.129	
**Sex**	0.11	-0.03	0.24	0.112		**0.18**	**0.05**	**0.31**	0.009*		**0.17**	**0.01**	**0.34**	0.039*		-0.01	-0.14	0.12	0.854	
**M10**	-0.15	-0.36	0.06	0.161		**-0.33**	**-0.51**	**-0.15**	< 0.001*		0.08	-0.09	0.25	0.359		0.20	-0.00	0.41	0.053	

β = standardized coefficient, CI = confidence intervals, *LL* = lower limit, *UL* = upper limit, R^2^ = Adjusted R-square for full model

Statistically significant associations (*p* < 0.05) are indicated in bold and with an *

*HTQ*, Harvard Trauma Questionnaire (1 best score), *PSQI* Pittsburgh Sleep Quality Index (1 best score), WHO-5 Well Being Index (100 best score), *M10*, mean activity score of the most active 10-hour period, measured in counts/hour; *SE* Sleep efficiency, measured in %

We found that the baseline level of physical activity was significantly associated with sleep quality (β = -0.33, *p* < 0.000). The baseline level of physical activity was borderline associated with baseline quality of life (β = 0.20, *p* = 0.053), but not significantly associated with the severity of PTSD symptoms, or sleep efficiency.

Table 4 presents the results of the multiple linear regression analyses on change values controlled for baseline score on the corresponding dependent variable, adjusted for age and sex. Change in the level of physical activity was significantly associated with improvement in sleep efficiency (β = 0.40, *p* = 0.002). The model accounted for 28% of the variance (R^2^) in treatment response for sleep efficiency. Change in the level of physical activity was not statistically significantly associated with improvement in the severity of PTSD symptoms, quality of life, or sleep quality.

**Table 4:** Multiple regression analyses of change in level of physical activity on change in PTSD symptoms (HTQ), quality of sleep (PSQI), quality of life (WHO-5), and sleep efficiency (SE) adjusted for baseline score, age and sex

	HTQ					PSQI					SE					WHO-5				
	β	95% CI for β		*p*	R^2^	β	95% CI for β		*p*	R^2^	β	95% CI for β		*p*	R^2^	β	95% CI for β		*p*	R^2^
		*LL*	*UL*				*LL*	*UL*				*LL*	*UL*				*LL*	*UL*		
					0.12					0.18					0.28					0.13
**Baseline score**	**0.29**	**0.12**	**0.44**	0.001*		**0.37**	**0.22**	**0.52**	< 0.0001*		**0.28**	**0.10**	**0.45**	0.002*		**0.28**	**0.12**	**0.043**	0.0001*	
**Age**	**-0.22**	**-0.37**	**-0.07**	0.005*		**-0.24**	**-0.42**	**-0.06**	0.008*		-0.06	-0.32	0.19	0.625		**0.23**	**0.06**	**0.40**	**0.006**	
**Sex**	0.00	-0.16	0.16	0.993		0.02	-0.14	0.14	0.978		-0.02	-0.26	0.22	0.866		-0.07	-0.24	0.09	0.373	
**M10**	-0.15	-0.54	0.24	0.452		-0.06	-0.26	0.31	0.868		**0.40**	**0.15**	**0.64**	0.002*		0.08	-0.23	0.38	0.613	

β = standardized coefficient, CI = confidence intervals, *LL* = lower limit, *UL* = upper limit, R^2^ = Adjusted R-square for full model

Statistically significant associations (*p* < 0.05) are indicated in bold and with an *

*HTQ*, Harvard Trauma Questionnaire (1 best score), *PSQI* Pittsburgh Sleep Quality Index (1 best score), WHO-5 Well Being Index (100 best score), *M10*, mean activity score of the most active 10-hour period, measured in counts/hour; *SE* Sleep efficiency, measured in %

The adjustment for age and sex lends to minor numerical changes in the estimates with no impact on the level of significance, nor overall conclusion. We tested for and found no confounding influences of employment in any of the models (data not shown).

## Discussion

The main findings of this study were that a higher daytime level of physical activity was significantly associated with better baseline sleep quality, borderline associated with quality of life but not with symptoms of PTSD, or sleep efficiency. Furthermore, change in the level of physical activity during the intervention added statistically significantly to the prediction of improvement in sleep efficiency. However, change in the level of physical activity did not add statistically significantly to the prediction of improvement in PTSD symptoms, quality of life, or sleep quality.

### Physical activity and symptoms of PTSD

This examination of the association between the level of physical activity and PTSD adds to a growing body of literature on the relation between the two [25, 29, 31]. The current study did not identify a significant relation between the baseline level of physical activity and symptoms of PTSD, replicating results from a review where half of the included studies reported no significant associations between PTSD and physical activity but diverging from the other half reporting a significant association between PTSD and physical activity [25]. The novelty of the current study lies in the finding of no relation between a change in the level of actigraphy measured physical activity and a change in symptoms of PTSD.

A previous study has shown a significant correlation between time spent walking and symptoms of PTSD, whereas the study did not find a correlation between self-reported moderate or vigorous physical activity and PTSD symptoms [45] possibly due to the limitations of self-report measures in comparison with technical measures of the intensity of physical activity. The current study lacks data on moderate or vigorous physical activity. In a previous publication, on the current sample, we highlighted the importance of leaving the house daily, contact with social networks, and exposure to daylight as important factors in relation to PTSD symptoms and circadian rhythm [46]. It is possible that the positive effect of time spent walking on symptoms of PTSD may also be related to leaving the house, contact with social networks, and exposure to daylight, and not merely the additional physical activity. Previous studies found no effect of physical activity interventions in trauma-affected refugees [22, 23].

One reason highlighted for the absence of effect of these interventions in refugees is a lack of previous experience with physical activity and unfamiliarity with the impact of physical activity on the body, such as pain in muscles after vigorous physical activity [22, 23]. We suggest further research to investigate the effect of interventions in trauma-affected refugees focusing on psychical activity in terms of both moderate and vigorous physical activity and on the role of activities enhancing both physical activity and activities outside the household, exposure to daylight and engagement in social networks. Furthermore, we suggest further research to study the effect of interventions in relation to both sexes and to include technical measures of sleep and physical activity [47].

### Physical activity and quality of life

A recent meta-analysis found a small but significant effect of physical exercise on the self-reported quality of life in populations with PTSD [20]. In the current study, we found an association between the baseline level of physical activity and quality of life, but did not identify a relation between an increase in the level of physical activity and an improvement in the quality of life. It is somewhat surprising that the baseline relation between the level of physical activity and quality of life is not replicated in the relation between the change in the level of physical activity and the change in the quality of life. However, it is in line with the prior mentioned study examining the effect of physical activity in trauma-affected refugees, which likewise found no intervention effect on quality of life [22].

### Physical activity and sleep parameters

In accordance with previous studies in PTSD populations, we found an association between higher physical activity levels and better baseline sleep quality [29, 31, 45, 48]. However, we did not identify a relation between an increase in the level of physical activity and an improvement in sleep quality. Contrary to these findings, while we identified no baseline relation between the level of physical activity and sleep efficiency, we found that an increase in the level of physical activity was significantly related to an increase in actigraphy measured sleep efficiency. The relation between a high level of physical activity and high sleep efficiency has previously been identified in the general population [30], but has, to our knowledge, not previously been studied in populations with PTSD, nor among refugees.

The cross-sectional design of both the current and previous studies does not allow for conclusions concerning the directionality of the relation. Physical activity may result in a higher sleep pressure and thus improve sleep, and poor sleep may cause daytime fatigue and less energy for physical activity [29].

Several meta-analyses have delineated interesting findings concerning the role of sleep in relation to PTSD [21, 49, 50]. A lower sleep efficiency has been identified in populations with PTSD compared to healthy controls, suggesting a promising effect of increasing sleep efficiency in order to improve PTSD symptoms [50]. The effect of exercise training on overall sleep quality among small samples of individuals with PTSD suggests that exercise training can improve overall sleep quality for those with PTSD [21], and a meta-analysis of the effects of physical activity on sleep in mixed populations found that regular exercise affected both sleep quality and sleep efficiency [49].

Current guidelines on the treatment of sleep disturbances in PTSD recommend cognitive-behavioral therapy for insomnia (CBT-I) [17], focusing on sleep cognitions, stabilizing sleep by sleep-wake schedule, including, among others, stimulus control strategies and regularity of rise- and bedtime to improve sleep efficiency. However, the identified relation between increase in the level of physical activity and an increase in sleep efficiency suggests an interesting shift in focus to daytime activity as a possible factor in interventions seeking to improve sleep efficiency.

Thus, the current findings support recommendations in our previous publication suggesting that interventions focusing on social rhythm regularity established by employment, activities encouraging leaving the house during the day and in daylight, as well as social networking, may likewise increase physical activity– and subsequently potentially also sleep efficiency [46]. Furthermore, regular daily activities may eliminate sleep-disruptive behaviors, such as late rising and napping, that interfere with the overall sleep process [51], and may strengthen sleep pressure and impact sleep efficiency and sleep quality.

### Strengths and limitations

The use of actigraphy to measure both physical activities and sleep efficiency is a key strength of the current study since self-report measures of physical activity may be impacted by recall bias, variability in the perception of activity intensity, and difficulty quantifying time spent being physically active [52]. Comparisons with previous studies on PTSD and physical activity that rely on self-report measures must be made with caution due to the differences in the methodological approach.

The equal proportions of men and women is a strength of the study due to underrepresentation of women in PTSD studies of military service members and veterans [25]. Furthermore, the use of longitudinal data is a strength. However, causal conclusions on the direction of the associations between change scores are not possible.

Actigraph-wearing compliance was low both at baseline- and at end of treatment, similar to that seen in other studies on populations with PTSD [53], posing a limitation. This limitation is also addressed in the method section. Forgetting to put on the actigraph after a short non-wear period was commonly reported. Feeling trapped and monitored was also noted as reasons, likewise was fear of stigma in the workplace or language school concerning the need for monitoring. Furthermore, it is a limitation that the actigraphy data included in this study does not distinguish between work or leisure time and does not provide knowledge regarding the intensity (low, moderate or vigorous) of the physical activity. Lastly, only small changes were identified from baseline to end of treatment posing a possible limitation.

## Conclusions

To our knowledge, this is the first study using actigraphy measures of physical activity in a longitudinal design in a population with PTSD. The study identified an association between an increase in physical activity and an increase in sleep efficiency, but not with improvement in symptoms of PTSD, quality of life, or sleep quality, in a sample of trauma-affected refugees. The relation between increase in the level of physical activity and an increase in sleep efficiency suggests an interesting shift in focus to daytime activity as a possible factor in interventions seeking to improve sleep efficiency.

The results point to a complex relation between sleep, physical activity and PTSD and a need for studies on these relations to provide effective interventions in trauma-affected refugees. We suggest further research on the effect of interventions on both symptoms of PTSD and sleep parameters, focusing on psychical activity in terms of interventions enhancing exercise and on the context for and the role of activities enhancing both physical activity and activities outside the house, exposure to daylight and engagement in social contact.

## Data Availability

The data supporting this study’s findings are available from the corresponding author on reasonable request.

## References

[1] Global Trends. Report 2022| UNHCR [Internet]. [cited 2023 Sep 17]. Available from: https://www.unhcr.org/global-trends-report-2022.

[2] UN Refugee Agency. UNHCR - Ukraine emergency [Internet]. 2022 [cited 2023 Feb 25]. Available from: https://www.unhcr.org/ukraine-emergency.html.

[3] United Nations. Convention and Protocol Relating to the Status of Refugees [Internet]. 1951 [cited 2020 Nov 10]. Available from: https://www.unhcr.org/3b66c2aa10. 12344222

[4] Steel Z, Chey T, Silove D, Marnane C, Bryant RA, van Ommeren M. Association of torture and other potentially traumatic events with mental health outcomes among populations exposed to mass conflict and displacement: a systematic review and meta-analysis. JAMA: The Journal of the American Medical Association. 2009;302(5):537–49. 19654388 10.1001/jama.2009.1132

[5] Blackmore R, Boyle JA, Fazel M, Ranasinha S, Gray KM, Fitzgerald G et al. The prevalence of mental illness in refugees and asylum seekers: a systematic review and meta-analysis. PLoS Med. 2020;17(9). 10.1371/journal.pmed.1003337 PMC7505461 32956381

[6] Giacco D, Priebe S. Mental health care for adult refugees in high-income countries. Epidemiology and Psychiatric sciences. Volume 27. Cambridge University Press; 2018. pp. 109–16. 10.1017/S2045796017000609 PMC6998959 29067899

[7] Silove D, Ventevogel P, Rees S. The contemporary refugee crisis: an overview of mental health challenges. World Psychiatry. 2017;16(2):130–9. 28498581 10.1002/wps.20438 PMC5428192

[8] Sijbrandij M. Expanding the evidence: key priorities for research on mental health interventions for refugees in high-income countries. Vol. 27, Epidemiology and Psychiatric sciences. Cambridge University Press; 2018. pp. 105–8. 10.1017/S2045796017000713 PMC6998957 29143713

[9] Carlsson J, Sonne C, Silove D. From pioneers to scientists: challenges in establishing evidence-gathering models in torture and trauma mental health services for refugees. J Nerv Ment Dis. 2014;202(9):630–7. 25167130 10.1097/NMD.0000000000000175

[10] Jou YC, Pace-Schott EF. Call to action: Addressing sleep disturbances, a hallmark symptom of PTSD, for refugees, asylum seekers, and internally displaced persons. Sleep Health [Internet]. 2022 Dec 1 [cited 2023 Jun 29];8(6):593–600. Available from: https://pubmed.ncbi.nlm.nih.gov/36511279/. 10.1016/j.sleh.2022.09.003 PMC9757843 36511279

[11] Turrini G, Purgato M, Acarturk C, Anttila M, Au T, Ballette F, et al. Efficacy and acceptability of psychosocial interventions in asylum seekers and refugees: systematic review and meta-analysis. Epidemiol Psychiatr Sci. 2019;28(04):376–88. 30739625 10.1017/S2045796019000027 PMC6669989

[12] Tribe RH, Sendt KV, Tracy DK. A systematic review of psychosocial interventions for adult refugees and asylum seekers. Journal of Mental Health. Volume 28. Taylor and Francis Ltd; 2019. pp. 662–76. 10.1080/09638237.2017.1322182 28485636

[13] Thompson CT, Vidgen A, Roberts NP. Psychological interventions for post-traumatic stress disorder in refugees and asylum seekers: a systematic review and meta-analysis. Clinical Psychology Review. Volume 63. Elsevier Inc.; 2018. pp. 66–79. 10.1016/j.cpr.2018.06.006 29936342

[14] Nosè M, Ballette F, Bighelli I, Turrini G, Purgato M, Tol W et al. Psychosocial interventions for post-traumatic stress disorder in refugees and asylum seekers resettled in high-income countries: systematic review and meta-analysis. PLoS ONE. 2017;12(2). 10.1371/journal.pone.0171030 PMC5289495 28151992

[15] Kip A, Priebe S, Holling H, Morina N. Psychological interventions for posttraumatic stress disorder and depression in refugees: a meta-analysis of randomized controlled trials. Clinical Psychology and Psychotherapy. Volume 27. John Wiley and Sons Ltd; 2020. pp. 489–503. 10.1002/cpp.2446 32191370

[16] Uphoff E, Robertson L, Cabieses B, Villalón FJ, Purgato M, Churchill R et al. An overview of systematic reviews on mental health promotion, prevention, and treatment of common mental disorders for refugees, asylum seekers, and internally displaced persons. Cochrane Database of Systematic Reviews. 2020;2020(9). 10.1002/14651858.CD013458.pub2 PMC8572368 32885850

[17] Colvonen PJ, Straus LD, Stepnowsky C, McCarthy MJ, Goldstein LA, Norman SB. Recent Advancements in Treating Sleep Disorders in Co-Occurring PTSD [Internet]. Vol. 20, Current Psychiatry Reports. Current Medicine Group LLC 1; 2018 [cited 2020 Mar 24]. p. 48. Available from: http://www.ncbi.nlm.nih.gov/pubmed/29931537. 10.1007/s11920-018-0916-9 PMC6645398 29931537

[18] Vancampfort D, Stubbs B, Richards J, Ward PB, Firth J, Schuch FB, et al. Physical fitness in people with posttraumatic stress disorder: a systematic review. Disabil Rehabil. 2017;39(24):2461–7. 27628485 10.1080/09638288.2016.1226412

[19] Rosenbaum S, Vancampfort D, Steel Z, Newby J, Ward PB, Stubbs B. Physical activity in the treatment of post-traumatic stress disorder: a systematic review and meta-analysis. Psychiatry Res. 2015;230(2):130–6. 26500072 10.1016/j.psychres.2015.10.017

[20] Björkman F, Ekblom Ö. Physical Exercise as Treatment for PTSD: a systematic review and Meta-analysis. Mil Med. 2022;187(9–10):e1103–13. 34850063 10.1093/milmed/usab497

[21] McGranahan MJ, O’Connor PJ. Exercise training effects on sleep quality and symptoms of anxiety and depression in post-traumatic stress disorder: a systematic review and meta-analysis of randomized control trials. Ment Health Phys Act. 2021;20:100385.

[22] Nordbrandt MS, Sonne C, Mortensen EL, Carlsson J. Trauma-affected refugees treated with basic body awareness therapy or mixed physical activity as augmentation to treatment as usual—A pragmatic randomised controlled trial. Martinuzzi A, editor. PLoS ONE. 2020;15(3):e0230300. 32163509 10.1371/journal.pone.0230300 PMC7067472

[23] Hasha W, Igland J, Fadnes LT, Kumar B, Haj-Younes J, Strømme EM et al. The Effect of Physiotherapy Group Intervention in Reducing Pain Disorders and Mental Health Symptoms among Syrian Refugees: A Randomized Controlled Trial. Int J Environ Res Public Health [Internet]. 2020 Dec 2 [cited 2022 Nov 5];17(24):1–15. Available from: https://pubmed.ncbi.nlm.nih.gov/33348794/. 10.3390/ijerph17249468 PMC7767069 33348794

[24] Nilsson H, Gustavsson C, Gottvall M, Saboonchi F. Physical activity, post-traumatic stress disorder, and exposure to torture among asylum seekers in Sweden: a cross-sectional study. BMC Psychiatry. 2021;21(1). 10.1186/s12888-021-03461-2 PMC8444359 34530806

[25] Hall KS, Hoerster KD, Yancy WS. Post-traumatic stress disorder, physical activity, and eating behaviors. Epidemiol Rev [Internet]. 2015 Jan 1 [cited 2022 Nov 6];37(1):103–15. Available from: https://pubmed.ncbi.nlm.nih.gov/25595169/. 10.1093/epirev/mxu011 25595169

[26] LeardMann CA, Kelton ML, Smith B, Littman AJ, Boyko EJ, Wells TS et al. Prospectively Assessed Posttraumatic Stress Disorder and Associated Physical Activity. Public Health Reports [Internet]. 2011 [cited 2023 May 27];126(3):371. Available from: /pmc/articles/PMC3072859/. 10.1177/003335491112600311 PMC3072859 21553666

[27] Chwastiak LA, Rosenheck RA, Kazis LE. Association of psychiatric illness and obesity, physical inactivity, and smoking among a national sample of veterans. Psychosomatics [Internet]. 2011 May [cited 2023 May 27];52(3):230–6. Available from: https://pubmed.ncbi.nlm.nih.gov/21565594/. 10.1016/j.psym.2010.12.009 PMC3094543 21565594

[28] Godfrey KM, Lindamer LA, Mostoufi S, Afari N. Posttraumatic stress disorder and health: a preliminary study of group differences in health and health behaviors. Ann Gen Psychiatry [Internet]. 2013 Sep 26 [cited 2023 May 27];12(1):30. Available from: /pmc/articles/PMC3852011/. 10.1186/1744-859X-12-30 PMC3852011 24070007

[29] Vancampfort D, Richards J, Stubbs B, Akello G, Gbiri CA, Ward PB, et al. Physical activity in people with posttraumatic stress disorder: a systematic review of correlates. J Phys Act Health. 2016;13(8):910–8. 27144877 10.1123/jpah.2015-0436

[30] Gubelmann C, Heinzer R, Haba-Rubio J, Vollenweider P, Marques-Vidal P. Physical activity is associated with higher sleep efficiency in the general population: the CoLaus study. Sleep. 2018;41(7). 10.1093/sleep/zsy070 29617980

[31] Chen A, Rosenbaum S, Wells R, Gould K, Ward PB, Steel Z. Obesity, physical activity and sleep quality in patients admitted to a posttraumatic stress inpatient ward. Australasian Psychiatry. 2020;28(3):270–3. 32391730 10.1177/1039856220917075

[32] Sandahl H, Jennum P, Baandrup L, Poschmann IS, Carlsson J. Treatment of sleep disturbances in trauma-affected refugees: study protocol for a randomised controlled trial. Trials. 2017;18(1). 10.1186/s13063-017-2260-5 PMC5674222 29110681

[33] Sandahl H, Jennum P, Baandrup L, Lykke Mortensen E, Carlsson J. Imagery rehearsal therapy and/or mianserin in treatment of refugees diagnosed with PTSD: Results from a randomized controlled trial. J Sleep Res [Internet]. 2021 [cited 2021 Mar 9]; Available from: https://pubmed.ncbi.nlm.nih.gov/33529449/. 10.1111/jsr.13276 PMC8365672 33529449

[34] Hollifield M, Warner TD, Lian N, Krakow B, Jenkins JH, Kesler J, et al. Measuring trauma and health status in refugees: a critical review. JAMA: The Journal of the American Medical Association. 2002;288(5):611–21. 12150673 10.1001/jama.288.5.611

[35] Mollica RF, Caspi-Yavin Y, Bollini P, Truong T, Tor S, Lavelle J. The Harvard Trauma Questionnaire. Validating a cross-cultural instrument for measuring torture, trauma, and posttraumatic stress disorder in indochinese refugees. J Nerv Ment Dis. 1992;180:111–6. 1737972

[36] Hollifield M. Measuring Trauma and Health Status in refugees: a critical review. JAMA: The Journal of the American Medical Association. 2002;288(5):611–21. 12150673 10.1001/jama.288.5.611

[37] Mollica RF, Caspi-Yavin Y. The Harvard Trauma Questionnaire: validating a cross-cultural instrument for measuring torture, trauma, and posttraumatic stress disorder in indochinese refugees. J Nerv Mental Disease. 1992;180(2):111–6. 1737972

[38] Blom EH, Bech P, Högberg G, Larsson JO, Serlachius E. Screening for depressed mood in an adolescent psychiatric context by brief self-assessment scales–testing psychometric validity of WHO-5 and BDI-6 indices by latent trait analyses. Health Qual Life Outcomes. 2012;10:149. 23227908 10.1186/1477-7525-10-149 PMC3575311

[39] Buysse DJ, Reynolds CF, Monk TH, Berman SR, Kupfer DJ. The Pittsburgh Sleep Quality Index: a new instrument for psychiatric practice and research. Psychiatry Res. 1989;28(2):193–213. 2748771 10.1016/0165-1781(89)90047-4

[40] Insana SP, Hall M, Buysse DJ, Germain A. Validation of the Pittsburgh Sleep Quality Index Addendum for posttraumatic stress disorder (PSQI-A) in U.S. male military veterans. J Trauma Stress. 2013;26(2):192–200. 23512653 10.1002/jts.21793 PMC3746481

[41] Gonçalves BSB, Adamowicz T, Louzada FM, Moreno CR, Araujo JF. A fresh look at the use of nonparametric analysis in actimetry. Sleep Medicine Reviews. Volume 20. W.B. Saunders Ltd; 2015. pp. 84–91. 10.1016/j.smrv.2014.06.002 25065908

[42] Ancoli-Israel S, Cole R, Alessi C, Chambers M, Moorcroft W, Pollak CP. The role of actigraphy in the study of sleep and circadian rhythms. Vol. 26, Sleep. Associated Professional Sleep Societies,LLC; 2003. p. 342–92. 10.1093/sleep/26.3.342 12749557

[43] Van Someren EJW, Swaab DF, Colenda CC, Cohen W, McCall WV, Rosenquist PB. Bright light therapy: improved sensitivity to its effects on rest- activity rhythms in Alzheimer patients by application of nonparametric methods. Chronobiol Int. 1999;16(4):505–18. 10442243 10.3109/07420529908998724

[44] Van Someren EJW. Improving actigraphic sleep estimates in insomnia and dementia: how many nights? J Sleep Res. 2007. 10.1111/j.1365-2869.2007.00592.x 17716276

[45] Rosenbaum S, Vancampfort D, Tiedemann A, Stubbs B, Steel Z, Ward PB, et al. Among inpatients, posttraumatic stress disorder symptom severity is negatively associated with time spent walking. J Nerv Mental Disease. 2016;204(1):15–9. 10.1097/NMD.0000000000000415 26558500

[46] Sandahl H, Baandrup L, Vindbjerg E, Jennum P, Carlsson J. Social zeitgebers and circadian dysrhythmia are associated with severity of symptoms of PTSD and depression in trauma-affected refugees. Eur Arch Psychiatry Clin Neurosci. 2021;271(7):1319–29. 33956223 10.1007/s00406-021-01266-8

[47] Sheppard-perkins MD, Malcolm SK, Hira SK, Smith SVM, Darroch FE. Exploring moderate to vigorous physical activity for women with post-traumatic stress disorder: a scoping review. Ment Health Phys Act [Internet]. 2022;23(August):100474.

[48] Talbot LS, Neylan TC, Metzler TJ, Cohen BE. The mediating effect of sleep quality on the relationship between PTSD and physical activity. J Clin Sleep Med. 2014;10(7):795–801. 25024659 10.5664/jcsm.3878 PMC4067445

[49] Kredlow MA, Capozzoli MC, Hearon BA, Calkins AW, Otto MW. The effects of physical activity on sleep: a meta-analytic review. J Behav Med. 2015;38(3):427–49. 25596964 10.1007/s10865-015-9617-6

[50] Lam L, Ho FYY, Wong VWH, Chan KW, Poon CY, Yeung WF et al. Actigraphic sleep monitoring in patients with post-traumatic stress disorder (PTSD): a meta-analysis. J Affect Disord. 2023;320. 10.1016/j.jad.2022.09.045 36174789

[51] Moss TG, Carney CE, Haynes P, Harris AL. Is daily routine important for sleep? An investigation of social rhythms in a clinical insomnia population. Chronobiol Int. 2015;32(1):92–102. 25187987 10.3109/07420528.2014.956361

[52] Liu F, Wanigatunga AA, Schrack JA. Review Assessment of Physical Activity in Adults Using Wrist Accelerometers. 10.1093/epirev/mxab004 PMC8900289 34215874

[53] Rosenbaum S, Tiedemann A, Sherrington C, Van Der Ploeg HP. Assessing physical activity in people with posttraumatic stress disorder: feasibility and concurrent validity of the International Physical Activity Questionnaire- short form and actigraph accelerometers. BMC Res Notes. 2014;7(1). 10.1186/1756-0500-7-576 PMC4167128 25164278

